# Clinical characteristics and impact of exchange transfusion in infant pertussis with extreme leukocytosis

**DOI:** 10.1186/s13052-025-01933-9

**Published:** 2025-03-18

**Authors:** Yuanyuan Wu, Chuan Gan

**Affiliations:** 1 Health Management Center, The Second Affiliated Hospital of Chongqing Medical University, Chongqing Medical University, Chongqing, China; 2 Department of Infectious Diseases Children’s Hospital of Chongqing Medical University, National Clinical Research Center for Child Health and Disorders, Ministry of Education Key Laboratory of Child Development and Disorders, Chongqing Key Laboratory of Child Rare Diseases in Infection and Immunity, The First Batch of Key Disciplines On Public Health in Chongqing, Chongqing, China

## Abstract

**Background:**

Extreme leukocytosis in pertussis is a rare condition, and without effective interventions to reduce white blood cell counts, the mortality rate can approach 100%. The clinical characteristics of these patients and the application of exchange transfusion (ET) in their management are not yet clear.

**Methods:**

This retrospective study examines the clinical characteristics and impact of ET in infant pertussis with extreme leukocytosis.

**Results:**

We have included six infant pertussis patients with extreme leukocytosis, all of whom were female and underwent ET. Two patients survived, while four died. The surviving patients were relatively older at disease onset compared to those who died, and all three unvaccinated patients died. All patients required admission to the pediatric intensive care unit, presenting with fever, whooping cough, cyanosis, severe pneumonia, and respiratory failure. Pulmonary consolidation, cardiovascular failure, and pulmonary hypertension (PH) were also common, especially among those who died. Hypoglycemia and seizures were rare. Acute-phase proteins, such as C-reactive protein and procalcitonin, were elevated to varying degrees. ET effectively reduced peripheral blood leukocytes; however, a significant increase in leukocytes was observed 1–2 days after the first ET in the deceased patients.

**Conclusion:**

Extreme hyperleukocytosis is more commonly observed in young female children with pertussis. Younger age, unvaccinated status, and the presence of concurrent heart failure and PH may be associated with a poor prognosis. ET can effectively reduce peripheral blood leukocytes, but a rapid leukocyte rebound post-ET may be indicative of impending death.

## Introduction

Pertussis, caused by *Bordetella pertussis*, is a significant health threat, particularly to children. It ranks as the fifth leading vaccine-preventable cause of infectious death in children worldwide [1]. Severe pertussis is an important cause of death in pertussis patients, with mortality rates ranging from 19.7 to 31% [2–3]. Most patients with severe pertussis are under three months of age, and this group accounts for the majority of deaths from this disease [4]. The clinical manifestations of severe pertussis typically include refractory hypoxemia, cardiogenic shock, and pneumonia associated with severe leukocytosis [5]. In young infants, death from severe pertussis is often linked to extreme leukocytosis with lymphocytosis, refractory pulmonary hypertension (PH), and cardiogenic shock [6–7]. Studies have demonstrated a clear correlation between white blood cell (WBC) counts exceeding 30 × 10^9/L and increased mortality in infants with pertussis [8]; when WBC counts exceed 50 × 10^9/L, the risk of death is nearly ten times higher than in patients with normal counts [9]. Extreme leukocytosis, defined as a WBC count exceeding 100 × 10^9/L, is a strong predictor of death in severe pertussis patients [10–11]. Without interventions to lower WBC counts, death is inevitable for those with WBC counts over 100 × 10^9/L [11–12]. Currently, treatment options for reducing WBC counts are limited, with exchange transfusion (ET) and leukapheresis being the primary methods [13]. Leukapheresis, due to its high technical requirements and the potential for severe adverse reactions during implementation, is rarely used in current clinical practice [14]. In contrast, ET is a common procedure in pediatric intensive care units (PICUs), especially for neonates, and rarely leads to serious complications [15]. Since Romano et al. [16] first reported the use of ET in severe pertussis cases in 2004, it has gained wider clinical acceptance due to its minimal complications and rapid reduction in leukocytosis burden. The clinical community has gradually recognized that ET can effectively reduce leukocytosis and improve the prognosis of patients with severe pertussis [2, 17]. Despite this, the use of ET in patients with extreme hyperleukocytosis and their prognosis remains poorly understood. Moreover, due to the rarity of extreme hyperleukocytosis in pertussis, the clinical characteristics of these patients have been underexplored. In this report, we discuss the clinical characteristics of patients with extreme leukocytosis in pertussis and the role of ET in their management.

## Methods

### Patient selection

This study included six patients diagnosed with pertussis at the Children’s Hospital of Chongqing Medical University between January 1, 2018, and October 31, 2024. All patients met the clinical manifestations of pertussis and were confirmed by PCR or culture methods, with peripheral blood WBC counts exceeding 100 × 10^9/L. Given that ET is an invasive procedure, informed consent was obtained from either the father or mother of each child before treatment. Indications for ET were based on the following criteria: (1) If the WBC count is above 30,000/mm^3^, the patient requires intensive care unit (ICU) monitoring. ET may be considered if the WBC count continues to rise and the patient develops respiratory failure, heart failure, or PH; (2) If the WBC count is above 40,000/mm^3^ and the patient has heart failure or echocardiographic evidence of PH; (3) If the WBC count is above 50,000/mm^3^ and the patient has respiratory failure and/or heart failure; (4) If WBC count is above 50,000/mm^3^ and the patient has respiratory failure and/or echocardiographic evidence of PH.

### Data collection

We collected demographic information, vaccination history, clinical symptoms, details of treatment process, and prognosis data of six pertussis patients who received ET. All data were retrieved from the electronic medical record system.

### Exchange transfusion procedure

For patients meeting ET criteria, peripheral arteriovenous access was established, including one arterial line and two venous lines (one arterial and one venous line for blood withdrawal, and one venous line for routine fluid infusion). Blood products, including concentrated red blood cell (RBC) suspension and plasma, were prepared in a 2:1 or 3:1 ratio, adjusted based on pre-ET hemoglobin and hematocrit values. ET was performed through peripheral arteriovenous access, with blood withdrawn via the arterial line and infused at the venous end. The exchange sequence was RBC suspension (half volume) → plasma → remaining RBC suspension. For every 100 ml of blood exchanged, 1 ml of calcium gluconate was continuously infused intravenously to prevent hypocalcemia. The ET rate was set at 60–80 ml/kg/h (gradually increasing), with a total ET volume of 80–100 ml/kg. During the process, heparin (1–5 units) was intermittently flushed through the arterial catheter every 50–60 ml of blood withdrawal to maintain catheter patency. Vascular access was regularly checked for coagulation, and heparin concentration was adjusted accordingly. Blood glucose, calcium levels, coagulation function, and blood pressure were monitored throughout the exchange process.

### Clinical indicators and related definitions

Vaccination history was obtained from vaccination records, with vaccination defined as having received at least one dose of the pertussis vaccine. In cases of suspected co-infection, blood and/or sputum cultures were collected to detect bacterial, *Mycoplasma*, or fungal infections. All patients underwent chest X-ray examination, and some received high-resolution computed tomography (HRCT) based on abnormal chest imaging. Pneumonia was diagnosed based on radiology reports, characterized by pulmonary infiltration or blurred lung images; pulmonary consolidation was defined as solidification changes in lung tissue, and atelectasis was diagnosed based on imaging showing lung collapse changes. Severe pneumonia was defined as radiological evidence of infiltration or blurred lung images accompanied by symptoms of respiratory failure. Respiratory failure was defined as clinical manifestations of hypoxemia (PaO_2_ < 60 mmHg) [18] and increased respiratory rate (< 1 year: > 60 breaths/minute, 1–3 years: > 50 breaths/minute). PH was diagnosed according to the European Society of Cardiology and European Respiratory Society standards [19], with echocardiographic evidence of increased pulmonary artery pressure. Heart failure was defined based on (1) clinical manifestations including cardiac dysfunction, pulmonary congestion, and systemic circulation congestion, (2) electrocardiographic monitoring indicating sinus tachycardia (< 1 year: > 160 beats/minute) [20], or (3) echocardiographic evidence of cardiac dysfunction.

## Results

### Demographic characteristics

Over a six-year period, six patients were diagnosed with extreme leukocytosis pertussis and underwent ET. Their demographic characteristics are shown in Table 1. All six patients were female, with two surviving and four deceased, resulting in a survival rate of 33.3% (2 out of 6). All three unvaccinated patients died. The deceased patients were younger at disease onset compared to the survivors, with three of the deceased patients being ≤ 60 days old. All patients were admitted to the PICU, with the time from disease onset to PICU admission ranging from a minimum of 4 days to a maximum of 10 days.

**Table 1:** Epidemiologic, clinical, laboratory features and treatment of 6 patients with extreme hyperleukocytosis pertussis

Case	Survive		Death			
	Patient 1	Patient 2	Patient 3	Patient 4	Patient 5	Patient 6
Age and sex	281-day-old girl	375-day-old girl	60-day-old girl	23-day-old girl	234-day-old girl	58-day-old girl
Birth weight(kg)	3.1	3.2	2.9	3.6	3.4	3
Vaccination status	3 dose of DTaP	1 dose of DTaP	Nonvaccinated	Nonvaccinated	3 dose of DTaP	Nonvaccinated
Admission to PICU	+	+	+	+	+	+
Time from onset of illness to admission to PICU(day)	7	7	5	10	4	6
Clinical evaluation on admission to PICU	Temperature 37.8ºC Heart rate 163 bpm Respiratory rate 93 bpm Blood pressure 92/58 mmHg Oxygen saturation 95% (nasal cannulae); a few fine moist rales	Temperature 36.8ºC Heart rate 156 bpm Respiratory rate 41 bpm Blood pressure 85/40 mmHg Oxygen saturation 92% (nasal cannulae); a few fine moist rales with a little wheeze	Temperature 38ºC Heart rate 192 bpm Respiratory rate 57 bpm Blood pressure 76/42 mmHg Oxygen saturation 91% (nasal cannulae); numerous fine moist rales with a few wheezes	Temperature 38.5ºC Heart rate 152 bpm Respiratory rate 30 bpm Blood pressure 96/73 mmHg Oxygen saturation 94% (CPAP); umerous fine moist rales	Temperature 36.5ºC Heart rate 180 bpm Respiratory rate 60 bpm Blood pressure 100/60 mmHg Oxygen saturation 90% (nasal cannulae); numerous fine moist rales with a few wheezes	Temperature 38.7ºC Heart rate 200 bpm Respiratory rate 46 bpm Blood pressure 62/36 mmHg Oxygen saturation 90% (nasal cannulae); numerous fine moist rales with a few wheezes
**Clinical symptoms and Complications**						
Axillary temperature(℃)	37.8	38	38	38.5	38.2	38.7
Whoop	+	+	+	+	+	+
Cyanosis	+	+	+	+	+	+
Hypoglycemia	0	0	+	0	0	0
Severe pneumonia	+	+	+	+	+	+
Respiratory failure	+	+	+	+	+	+
Pulmonary consolidation	0	+	+	+	+	+
Cardiovascular failure	+	0	0	+	+	+
Pulmonary hypertension	0	0	0	+	+	+
Seizure	+	0	0	0	0	0
**Laboratory index**						
Raised CK-MB	0	0	+	+	+	0
Raised ALT	+	0	0	0	0	0
Acute-phase proteins	CRP 31 mg/dL PCT 0.898 ng/mL	CRP 15 mg/dL PCT 0.182 ng/mL	CRP 29 mg/dL PCT 0.218 ng/mL	CRP 25 mg/dL PCT 47.56 ng/mL	CRP 32 mg/dL PCT 0.78 ng/mL	CRP 28 mg/dL PCT 1.1 ng/mL
Echocardiogram	Normal	Normal	Not done	Right ventricle and atrium dilation and Moderate pulmonary hypertension.	Right ventricle and atrium dilation, mild pulmonary hypertension, and reduced left ventricular diastolic function.	Severe pulmonary hypertension, reduced left ventricular diastolic function.
Chest radiograph	Bilateral pneumonia	Bilateral pneumonia with multi-lobar consolidation	Bilateral pneumonia with multi-lobar consolidation	Bilateral pneumonia with partial consolidation	Bilateral pneumonia with segmental consolidation in both upper lobes	Bilateral pneumonia with associated pulmonary consolidation and atelectasis
Etiologic diagnosis and Coinfections	PCR positive for Bordetella pertussis; Sputum culture shows Haemophilus influenzae	PCR positive for Bordetella pertussis; ICG test positive for RSV (on admission) and Sputum culture shows Chryseobacterium indologenes	PCR positive for Bordetella pertussis; Sputum culture shows Klebsiella pneumoniae	PCR positive for Bordetella pertussis; Sputum culture shows Escherichia coli	PCR positive for Bordetella pertussis; Sputum culture shows Moraxella catarrhalis	PCR positive for Bordetella pertussis; Sputum culture shows Klebsiella pneumoniae
**Treatment**						
Immunoglobulin therapy	+	0	0	+	0	+
Corticosteroid therapy	+	0	0	+	0	0
Macrolide treatments	+	+	+	+	+	+
Time of macrolide use(Day)	7	5	3	4	3	5
Antibiotic treatment	Intravenous azithromycin + intravenous cefotaxime	Intravenous azithromycin + intravenous Meropenem	Intravenous azithromycin + intravenous Imipenem and Cilastatin Sodium for Injection	Intravenous azithromycin + intravenous Meropenem	Intravenous azithromycin + intravenous Imipenem and Cilastatin Sodium for Injection + intravenous cefotaxime	Intravenous azithromycin + intravenous Meropenem + intravenous cefotaxime

### Clinical characteristics, laboratory tests, imaging, and treatment

The clinical characteristics, laboratory findings, and imaging results of the patients are shown in Table 1. All patients exhibited common clinical manifestations, including fever, whooping cough, cyanosis, severe pneumonia, and respiratory failure. Pulmonary consolidation, cardiovascular failure, and PH were also relatively common, especially in patients who died. However, hypoglycemia and seizures were not common. Acute-phase proteins, such as C-reactive protein (CRP) and procalcitonin (PCT), were elevated to varying degrees. Organ function markers showed that increased creatine kinase-MB (CK-MB) was observed only in the deceased patients, while abnormal ALT levels were not found in any deceased patient. In terms of concurrent infections, all patients had bacterial co-infections, with Gram-negative bacilli being the predominant pathogens. All patients received azithromycin and other antibiotics. Three patients received immunoglobulin, and two received steroids.

### Exchange transfusion

The details of the ET treatment are shown in Table 2. All six patients underwent ET, with four patients receiving a second ET. The time from disease onset to the first ET varied, ranging from 5 to 19 days. The ET volumes for surviving patients were 1.12 and 1.18 times the blood volume, while for deceased patients, the volumes were 1.44, 1.32, 1.03, and 0.94 times the blood volume. The transfusion rate was higher in deceased patients than in survivors. The decrease in WBC count after the first ET was more pronounced in deceased patients than in surviving patients. The lymphocyte-to-neutrophil ratio was higher in the two surviving patients (1.78 and 3.13) compared to the deceased patients (0.49, 0.58, 1.04, and 1.07).

**Table 2:** Exchange transfusion

Case	Survive		Death			
	Patient 1	Patient 2	Patient 3	Patient 4	Patient 5	Patient 6
Time of ET	2	1	2	2	2	1
Time from onset of illness to ET(Day)	8	19	5	10	6	14
ET details	675 mL, Includ 575 ml of Packed RBCs and 100 ml of plasma; 1.12 times the blood volume in 3 h	900 mL, Includ 600 ml of Packed RBCs and 300 ml of plasma; 1.18times the blood volume in 2 h	600 mL, Includ 400 ml of Packed RBCs and 200 ml of plasma; 1.44times the blood volume in 1 h20min	450 mL, Includ 300 ml of Packed RBCs and 150 ml of plasma; 1.32 times the blood volume in 1 h30min	700 mL, Includ 400 ml of Packed RBCs and 300 ml of plasma; 1.03times the blood volume in 2 h20min	400 mL, Includ 300 ml of Packed RBCs and 100 ml of plasma; 0.94 times the blood volume in 1 h20min
The ratio of Packed RBCs to plasma	5.75: 1	2: 1	2: 1	2: 1	1.33: 1	3: 1
ET rate (ml/kg.h)	31.69	50	91.84	84.37	58.33	60
WBC count preprocedure(*10^9/L)	107.25	82.87	80.21	123.23	147.16	100.11
WBC count postprocedure(*10^9/L)	58.29	38.57	26.16	47.72	66.83	37.29
Leukocyte count decreases post First ET(*10^9/L)	48.96	44.3	54.05	75.51	80.33	62.82
Lymphocyte/Neutrophil ratio	1.78	3.13	0.49	0.58	1.04	0.63

### Peripheral blood and its changes

**Fig. 1:**
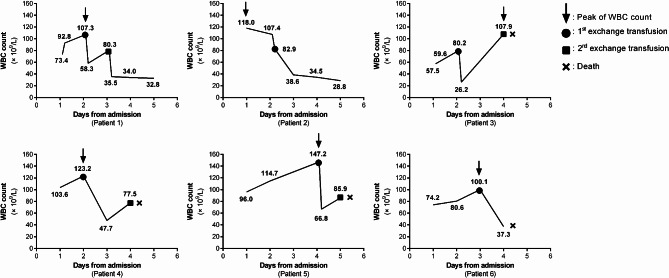
Peripheral white blood cell count changes in six pertussis patients with leukocytosis undergoing exchange transfusion

The changes in peripheral blood WBC count for all six patients are shown in Fig. 1. In the first patient, WBC count significantly decreased after the first ET but increased again within 24 h, no further increase was observed after the second ET. The second patient showed a significant decrease in WBC after the first ET, with no subsequent increase. Patients 3 to 5 also showed a significant decrease in WBC after the first ET, but all had a noticeable increase within 1–2 days and died after the second ET, suggesting that a significant rebound in WBC after ET may be indicative of poor prognosis. The sixth patient had a significant decrease in WBC after the first ET but still died due to circulatory failure.

## Discussion

Pertussis, a highly contagious and vaccine-preventable disease, has experienced a considerable decrease in incidence due to extensive immunization programs over the past several decades. In 2018, the World Health Organization reported 151,074 cases of pertussis, with a mortality rate of 4% [21]. A considerable proportion of these deaths occurred in severe cases complicated by hyperleukocytosis. Currently, it is widely accepted that the mortality rate for pertussis patients with extreme hyperleukocytosis approaches 100%. In our study, the survival rate with ET was 33.3%, which is higher than in previous studies. This may be due to the effective reduction of peripheral leukocytes and leukocyte toxins by ET, which helps reduce pulmonary capillary occlusion, and thereby improving PH and preventing progression to cardiogenic shock [14, 22–23]. In our study, all patients experienced a significant decrease in leukocytes, with deceased patients showing a more pronounced decrease. This suggests that while ET can significantly reduce peripheral blood leukocytes, it cannot solely determine the patient’s prognosis. Close monitoring of peripheral blood cells is necessary, as a significant increase in leukocytes within 1–2 days after ET is highly indicative of mortality, even when repeated ET is performed.

Gender may play a role in the occurrence and progression of certain diseases, with studies suggesting that the risk of mortality in severe pertussis is higher in females than in males [24]. While our study included only female patients, this does not imply that only females can develop extreme leukocytosis, but it does highlight that the incidence in females may be higher.

Pertussis vaccination is a crucial preventive measure against severe disease and has been widely implemented globally, particularly in China. Over the past decade, the vaccination coverage for three doses of the DTP vaccine in China has consistently remained above 99%. In China, infants under three months of age cannot receive their first pertussis vaccine, and most severe pertussis cases occur in infants under this age [4]. This increases the risk of infection and often results in more severe disease. Our study supports this view, as all three unvaccinated patients were ≤ 60 days old, and all died.

Acute-phase proteins such as CRP and PCT are indicators of systemic inflammation, often elevated together with body temperature in the presence of concurrent infections. In our study, all patients exhibited increased temperatures, and acute-phase proteins were elevated to varying degrees. This suggests that concurrent infection is common in pertussis patients with extreme leukocytosis, which aligns with our finding of bacterial co-infections in all patients. Liver dysfunction, as indicated by abnormal ALT levels, was uncommon in these patients, contrasting with elevated CK-MB in those who died, potentially reflecting cardiac dysfunction.

Severe pertussis is reported to be associated with complications such as refractory hypoxemia (caused by severe pneumonia), PH, and cardiogenic shock [8, 25]. Consistently, our study found that patients with extreme leukocytosis were prone to these complications. Pulmonary consolidation, cardiovascular failure, and PH were common, particularly in those who died.

Hyperleukocytosis can lead to increased blood viscosity and pulmonary vascular resistance, potentially resulting in PH and hemodynamic collapse, with death often due to hypoxemia and refractory shock. Leukocytosis in severe pertussis may be due to the pertussis toxin (PT) [8]. Although the mechanism by which PT induces leukocytosis remains not fully elucidated [22], it is a significant feature in clinical patients with PH. Furthermore, extreme leukocytosis (WBC > 100 × 10^9/L) is strongly associated with poor prognosis [26]. In our study, 50% (3/6) of patients exhibited PH, and 66.7% (4/6) had cardiovascular failure. Notably, all three patients who presented with both PH and cardiovascular failure died, suggesting that the co-occurrence of these conditions significantly increases the risk of mortality. However, we observed that not all patients with extreme leukocytosis developed PH or cardiac failure, indicating that the presence of PH and other conditions may not be entirely dependent on peripheral leukocyte counts.

Since Romano et al. [16] first reported the use of ET for severe pertussis in 2004, its clinical efficacy in reducing leukocyte levels has been well-established [2, 15, 17]. Our study also confirmed that ET can effectively reduce peripheral blood leukocytes in all patients. However, although leukocytes significantly decreased after the first ET, most patients experienced a rebound, especially those who died. This emphasizes the importance of closely monitoring leukocyte counts after ET, as a significant rebound may necessitate a second or multiple ET, consistent with previous studies [12, 27–28].

Although ET has become more widely used, the volume of exchange is not standardized, with 1 times [15], 1.5 times [14], and 2 times [16] of blood volumes reported. In our study, the volume of exchange was approximately 1 to 1.5 times the blood volume. There are few studies reported exchange rates, our study found that the ET rate in deceased patients all exceeded 50 ml/kg/h, while in survivors, it did not. Although this is an interesting phenomenon, our study cannot definitively conclude that this rate is a factor affecting patient outcomes. Similarly, although the decrease in leukocytes after the first ET was similar, deceased patients experienced a reduction exceeding 50 × 10^9/L, while surviving patients did not. This may suggest that a rapid leukocyte reduction may contribute to mortality, but further studies are required to confirm this.

## Conclusions

Our clinical study suggests that patients with extreme leukocytosis in pertussis are predominantly young female children. Younger age, unvaccinated status, the presence of concurrent PH and cardiac failure, and a rapid re-elevation of WBC after the first ET are associated with a poor prognosis. These patients are also highly susceptible to bacterial infections, which may have contributed to the exacerbation of their conditions. Although our study is based on a small sample size from a single center, it provides valuable insights into the clinical characteristics of these patients and the potential risks and benefits of ET treatment. Larger, multi-center, and prospective studies are needed to further investigate the clinical features of these patients and better evaluate the effectiveness of ET treatment.

## Data Availability

The datasets used and/or analyzed during the current study are available from the corresponding author on reasonable request.

## Abbreviations

CRP: C-reactive protein
ET: Exchange transfusion
PCT: Procalcitonin
PH: Pulmonary hypertension
RBC: Red blood cell
WBC: White blood cell
